# Supplementary material for: A Non-Functional Carbon Dioxide-Mediated Post-Translational Modification on Nucleoside Diphosphate Kinase of Arabidopsis thaliana
Source: Int J Mol Sci. 2024 Jan 11;25(2):898. doi: 10.3390/ijms25020898 (PMC10815852; doi:10.3390/ijms25020898)
Supplement: Supplementary file 1 [file ijms-25-00898-s001.zip › ijms-2793831-supplementary.pdf]

A.

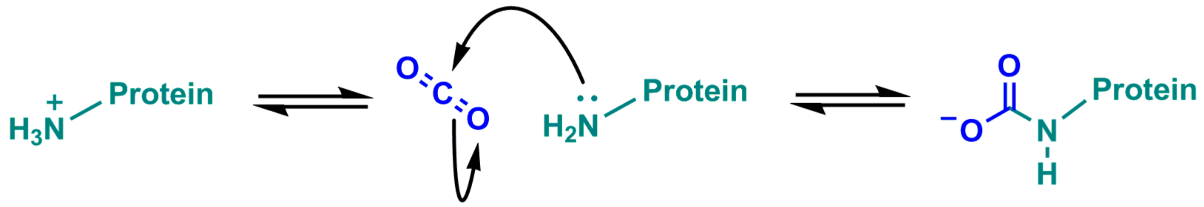

B.

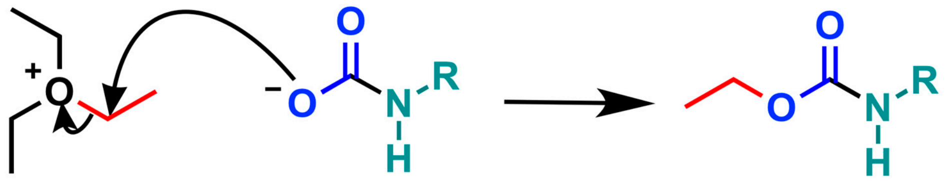

Supplementary Figure S1. A. Carbamates form through the reversible reaction between CO_2 and neutral amine groups**. B**. Trapping a protein carbamate with TEO. TEO transfers an ethyl group (red) to the anionic carbamate derived from CO_2 (blue) and protein primary amine (green).

A

```
NDK1_ARATH         1 ---MEQTFIMIKPDGVQRGLIGEVICRFEKKGFTLKGLKLISVERSFAEK     47
                        .|:|||.||||||||||:||:|.|||:|||.|.|||.:.......::
NDKA_HUMAN         1 MANCERTFIAIKPDGVQRGLVGEIIKRFEQKGFRLVGLKFMQASEDLLKE     50

NDK1_ARATH        48 HYEDLSSKSFFSGLVDYIVSGPVVAMIWEGKNVVLTGRKIIGATNPAASE     97
                     ||.||..:.||:|||.|:.|||||||:|||.|||.|||.::|.||||.|:
NDKA_HUMAN        51 HYVDLKDRPFFAGLVKYMHSGPVVAMVWEGLNVVKTGRVMLGETNPADSK    100

NDK1_ARATH        98 PGTIRGDFAIDIGRNVIHGSDSVESARKEIALWF-PDGPVNWQSSVHPWV    146
                     ||||||||.|.:|||:||||||||||.|||.||| |:..|::.|....|:
NDKA_HUMAN       101 PGTIRGDFCIQVGRNIIHGSDSVESAEKEIGLWFHPEELVDYTSCAQNWI    150

NDK1_ARATH       147 YET    149
                     ||
NDKA_HUMAN       151 YE-    152
```

(In the printed alignment, the K at position 12 of NDK1_ARATH and of NDKA_HUMAN is in bold and underlined.)

B

$$\mathrm{XTP} + \mathrm{YDP} \;\rightleftharpoons\; \mathrm{XDP} + \mathrm{YTP} \qquad (1)$$

$$\mathrm{NDK} + \mathrm{XTP} \;\rightleftharpoons\; \mathrm{NDK} - \mathrm{XTP} \;\rightleftharpoons\; \mathrm{NDK} \cdot \mathrm{P} - \mathrm{XDP} \;\longrightarrow\; \mathrm{NDK} \cdot \mathrm{P} + \mathrm{XDP} \quad (2)$$

$$\mathrm{NDK} \cdot \mathrm{P} + \mathrm{YDP} \;\rightleftharpoons\; \mathrm{NDK} \cdot \mathrm{P} - \mathrm{YDP} \;\rightleftharpoons\; \mathrm{NDK} - \mathrm{YTP} \;\rightarrow\; \mathrm{NDK} + \mathrm{YTP} \quad (3)$$

C

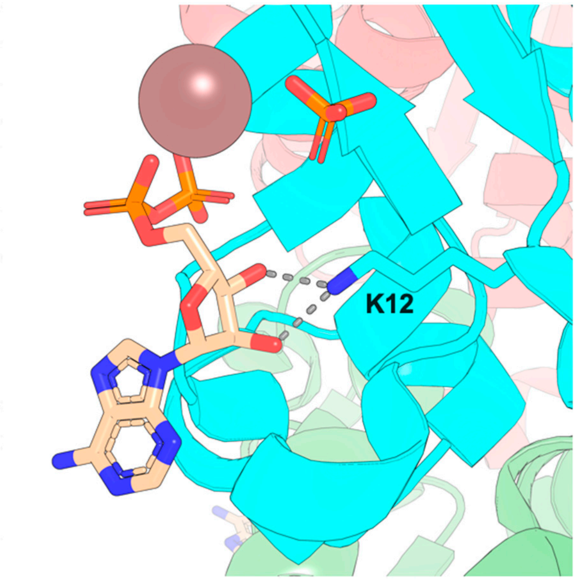

Supplementary Figure S2. A. Protein sequence alignment calculated using the Needleman-Wunsch algorithm for the ApcA open reading frames of *Arabidopsis* NDK1 (NDK1_ARATH) and human NDK1 (NDKA_HUMAN). Number indicates amino acid number, . (period) indicates conservation between groups of strongly similar properties (>0.5 in the Gonnet PAM 250 matrix), : (colon) indicates conservation between groups of weakly similar properties (=< 0.5 in the Gonnet PAM 250 matrix). The carbamylated lysines are shown in bold and underlined. **B.** NDKs catalyse the reversible transfer of the γ-phosphoryl group of a nucleoside triphosphate to a nucleoside diphosphate (the reaction from which they derive their name). The overall reaction is given by equation 1, and the phospho-transfer half-reactions are given by equations 2 and 3. **C.** Ribbon diagram of the x-ray crystal structure of human NDK1 (Protein Data Bank code 1UCN) showing the secondary and tertiary structure of the region of NDK1 with ADP bound. The inset shows the potential hydrogen-bonding network between ADP and K12. The three chains of PDB 1UCN are coloured cyan/salmon/light green, ADP in wheat and the calcium atom in dark purple. O/N/P are shown as red/blue/orange as per standard. Two alpha-helices over the site, from E45-S70, are hidden for clarity.

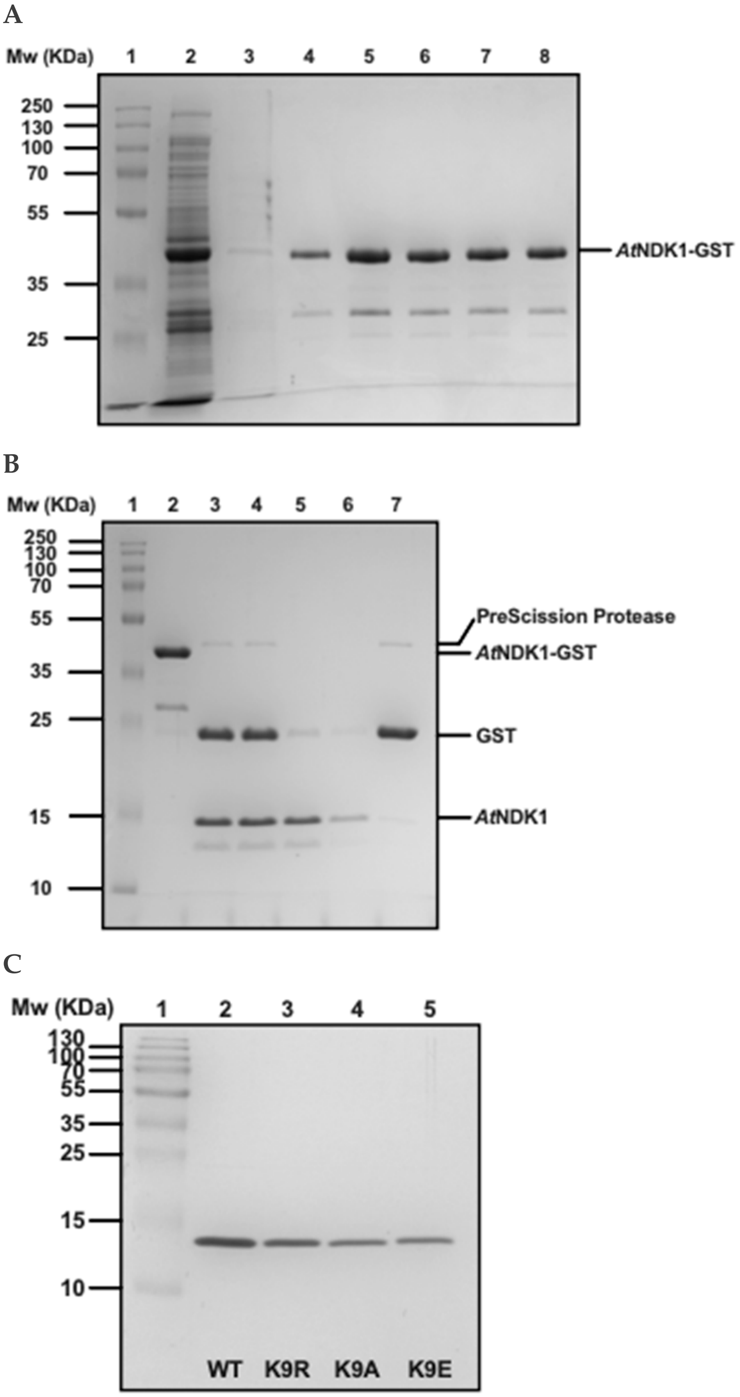

Supplementary Figure S3. A. Example of SDS/PAGE analysis and Coomassie Blue staining showing *At*NDK1 GST fusion protein purification. Lanes are 1. Molecular mass standards; 2. Flow through; 3. Wash; 4-7. Reduced glutathione elutions. **B.** Example of SDS/PAGE analysis and Coomassie Blue staining showing *At*NDK1 GST fusion protein cleavage. Lanes are 1. Molecular mass standards; 2. *At*NDK1 GST fusion protein sample; 3-4. *At*NDK1 GST fusion protein sample post-cleavage; 5. Flow through; 6. Wash; 7. Reduced glutathione elution. **C**. Example of SDS/PAGE analysis and Coomassie Blue staining showing final *At*NDK1 wild type and mutant purified recombinant proteins. Lanes are 1. Molecular mass standards; 2. *At*NDK1-WT; 3. *At*NDK1-K9R; 4. *At*NDK1-K9A; 5. *At*NDK1-K9E.

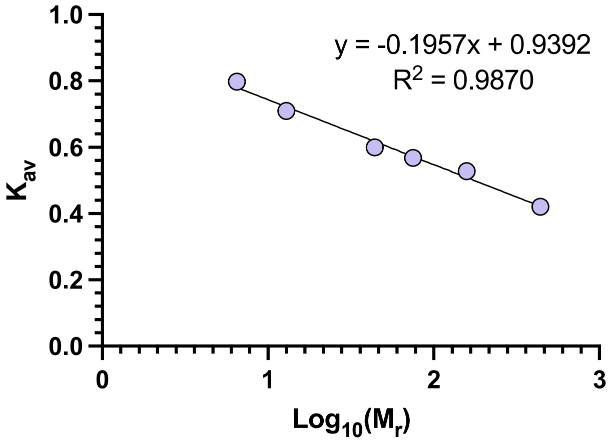

Supplementary Figure S4. Plot of partition coefficient (K_{av}) against log protein molecular weight ($Log_{10}(M_r)$) for known protein standards.

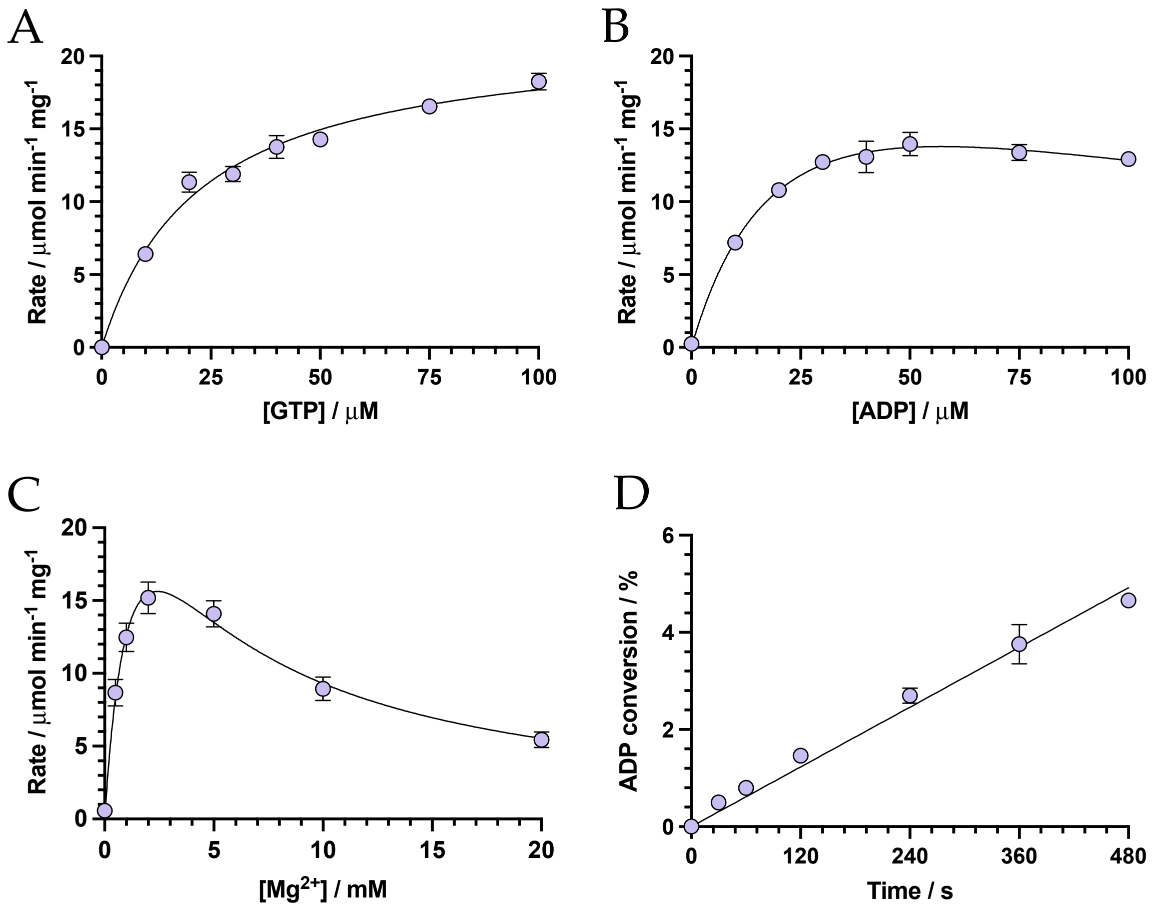

Supplementary Figure S5. Biochemical characterisation of AtNDK1. **A.** *At*NDK1 activity rate plotted against variable [GTP] at fixed 35 μM ADP. Each point represents mean ± S.E.M, *n* = 3. **B.** *At*NDK1 activity rate plotted against variable [ADP] at fixed 50 μM GTP. Each point represents mean ± S.E.M, *n* = 3. **C.** *At*NDK1 activity rate plotted against [Mg^{2+}] at 50 μM GTP and 35 μM ADP. Each value represents mean ± S.E.M, n = 3. **D.** % ADP conversion plotted against time to determine the linear range of the NDK assay under the determined standard assay conditions. Each point represents mean ± S.E.M, *n* = 3. r^2 = 0.9945.
